# Anomalous Left Coronary Artery from the Pulmonary Artery: Cinematic Volume Rendering Technique for Enhanced Anatomic Visualization

**DOI:** 10.3390/diagnostics16121940

**Published:** 2026-06-22

**Authors:** Shuo Liang, Kun Zhang, Hong Zhang

**Affiliations:** Department of Radiology, Chest Hospital, Tianjin University, Tianjin 300222, China; movingspirit@163.com (S.L.); 18803423746@163.com (K.Z.)

**Keywords:** anomalous left coronary artery from the pulmonary artery, coronary CT angiography, cinematic volume rendering technique, coronary anomaly, congenital heart disease

## Abstract

Anomalous left coronary artery from the pulmonary artery (ALCAPA) is a rare congenital anomaly with exceptional survival into adulthood. We present a 66-year-old woman with chest and back pain in whom ALCAPA was diagnosed using coronary computed tomography angiography (CCTA) with curved planar reformation and cinematic volume rendering technique (cVRT). Photorealistic three-dimensional reconstruction provided complementary three-dimensional visualization that may facilitate anatomic understanding and communication of the anomalous origin. Conservative management was adopted given the patient’s age and well-developed collateral circulation. This case underscores the value of advanced CCTA visualization in diagnosing rare coronary anomalies in elderly patients.

**Figure 1:**
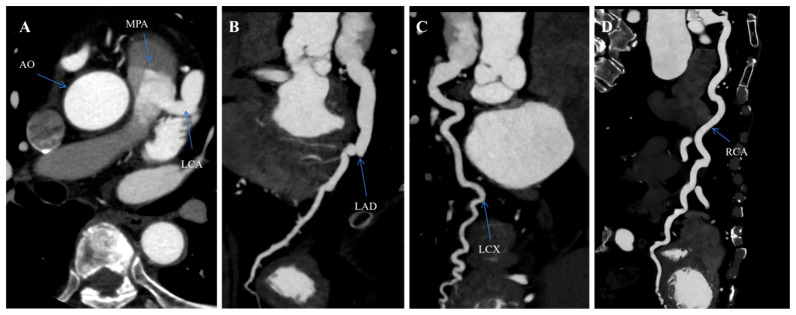
A 66-year-old woman presenting with intermittent chest pain and upper back pain for three months. (**A**) Axial coronary CTA image demonstrates anomalous origin of the left coronary artery (LCA; arrow) from the main pulmonary artery (MPA; arrow), with high-density contrast medium shunting into the MPA. Curved planar reformation (CPR) images clearly delineate the full course of the anomalous vessel from its anomalous ostium in the MPA, coursing along the expected left atrioventricular groove, giving rise to the LAD ((**B**); arrow) and LCX ((**C**); arrow). The proximal LAD shows marked luminal dilatation. (**D**) The right coronary artery (RCA; arrow) is significantly dilated and tortuous. The high spatial resolution of CPR enables precise evaluation of the anomalous ostial location, vessel caliber, and collateral development—information that is valuable for anatomic understanding and communication among the multidisciplinary team in adult ALCAPA patients.

**Figure 2:**
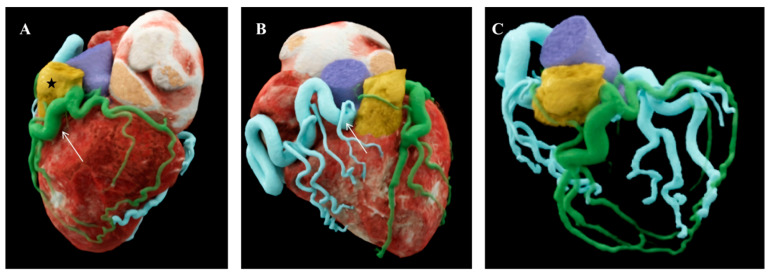
Cinematic volume rendering technique (cVRT) provides photorealistic three-dimensional visualization of the anomalous coronary anatomy. Unlike conventional volume rendering techniques, cVRT employs advanced complex lighting models that simulate real-world illumination conditions, producing natural shadows and enhancing surface detail representation. This technique significantly improves depth perception and tissue discrimination, rendering the anomalous origin and the interarterial course readily apparent. The photorealistic imaging quality of cVRT facilitates effective multidisciplinary communication and anatomic information presentation during patient counseling. (**A**) An anterior oblique view shows the anomalous origin of the left coronary artery (arrow) from the main pulmonary artery (asterisk). (**B**) A posterolateral view clearly demonstrates the spatial relationship among the anomalous vessel, the pulmonary trunk, and the aortic root. The right coronary artery (arrow) appears dilated and dominant. (**C**) The RCA provides retrograde perfusion to the LCA territory through extensive intercoronary collateral vessels. Anomalous left coronary artery from the pulmonary artery (ALCAPA), also known as Bland-White-Garland syndrome, is a rare congenital coronary anomaly with an estimated incidence of approximately 1 in 300,000 live births, accounting for 0.24% to 0.46% of all congenital cardiac defects [1]. The anomaly is characterized by the anomalous origin of the left coronary artery from the pulmonary artery rather than the left aortic sinus. In the neonatal period, when pulmonary vascular resistance falls, perfusion of the anomalous left coronary artery becomes inadequate, leading to myocardial ischemia, infarction, and heart failure. Without surgical intervention, approximately 90% of affected infants succumb within the first year of life [2]. Survival into adulthood represents an exceptional phenomenon, occurring in fewer than 10% of cases, and is predicated on the development of robust intercoronary collateral circulation from a dominant right coronary artery [3]. Among adult survivors, a female predominance exceeding 2:1 has been consistently observed [2,4]. Adult presentations span a broad clinical spectrum, ranging from incidental discovery in asymptomatic individuals to exertional angina, dyspnea, malignant arrhythmias, syncope, or sudden cardiac death [4]. We present the case of a 66-year-old woman who presented to our cardiology department with intermittent chest and upper back pain of three months’ duration. The pain was non-exertional, occurred predominantly at rest, and was partially responsive to sublingual nitroglycerin. The patient had a remote history of hypertension controlled with antihypertensive therapy. Physical examination revealed unremarkable cardiovascular findings. Resting electrocardiography demonstrated T-wave inversions in leads I, aVL, and V4 through V6, suggestive of chronic lateral wall ischemia. Transthoracic echocardiography showed preserved left ventricular ejection fraction with akinesia of the basal anterolateral segment. Given the atypical symptomatology and electrocardiographic abnormalities in an elderly patient, CCTA was performed to exclude coronary artery disease and evaluate the coronary anatomy. CCTA was performed on a third-generation dual-source CT scanner (Siemens Healthineers, Forchheim, Germany) following institutional protocol. Acquisition parameters included retrospective electrocardiographic gating with a slice thickness of 0.75 mm, tube voltage of 100 kV, and automated tube current modulation. Intravenous iodinated contrast (370 mg I/mL) was administered at 5 mL/s, followed by a saline chaser. Curved planar reformation (CPR) and cinematic volume rendering technique (cVRT) were generated using dedicated post-processing software. CCTA revealed the left coronary artery arising anomalously from the left posterolateral aspect of the main pulmonary artery, 1.2 cm above the pulmonary valve (Figure 1). The anomalous vessel followed the expected course of the left coronary system along the left atrioventricular groove. The left main coronary artery measured 3.2 mm in diameter, terminating into the left anterior descending and circumflex arteries. The right coronary artery was markedly dilated, measuring 6.8 mm at its mid-segment, with prominent tortuosity and extensive collateral vessels communicating with the distal left coronary system, consistent with the adult or collateral-dependent subtype of ALCAPA [5]. No significant atherosclerotic stenosis was identified in either coronary system. The aorta and remaining thoracic vasculature were unremarkable. cVRT reconstructions provided photorealistic three-dimensional visualization that facilitated intuitive comprehension of the anomalous origin and its spatial relationship with adjacent mediastinal structures (Figure 2). Compared with conventional volume rendering, cVRT employs sophisticated illumination algorithms incorporating complex light-path simulations, producing naturalistic shadows, enhanced surface texture detail, and substantially improved depth perception, thereby enabling enhanced anatomic delineation for both diagnostic interpretation and interdisciplinary consultation [6]. These advanced visualization techniques collectively enable enhanced anatomic understanding that may facilitate communication among the multidisciplinary team. These visualization capabilities are particularly valuable for conveying complex coronary anomalies to referring clinicians and for preprocedural surgical planning. The diagnosis of ALCAPA in this patient was established non-invasively by CCTA. The patient had a remote history of hypertension controlled with antihypertensive therapy. Serum cardiac troponin I was mildly elevated at 0.08 ng/mL (reference < 0.04 ng/mL), and N-terminal pro-B-type natriuretic peptide was 245 pg/mL (reference < 125 pg/mL). Stress myocardial perfusion imaging was not performed given the patient’s frailty and substantial anxiety. Given the atypical chest pain presentation with chronic ischemic electrocardiographic changes in an elderly patient, CCTA was selected as the initial non-invasive imaging modality to simultaneously evaluate coronary anatomy and exclude atherosclerotic disease. CCTA was performed on a third-generation dual-source CT scanner (SOMATOM Force, Siemens Healthineers, Forchheim, Germany) following institutional protocol. Acquisition parameters included retrospective electrocardiographic gating with a slice thickness of 0.75 mm, tube voltage of 100 kV, and automated tube current modulation. A total of 85 mL of intravenous iodinated contrast (Iopromide, 370 mg I/mL; Ultravist, Bayer HealthCare, Berlin, Germany) was administered at 5 mL/s via an 18-gauge antecubital intravenous catheter, followed by a 50 mL saline chaser using a dual-head power injector (Ulrich GmbH & Co. KG, Ulm, Germany). CPR and cVRT reconstructions were generated using syngo.CT Coronary Analysis and syngo.via Cinematic VRT software (syngo.via VB40A, Siemens Healthineers, Forchheim, Germany), respectively. CCTA revealed the left coronary artery arising anomalously from the left posterolateral aspect of the main pulmonary artery, 1.2 cm above the pulmonary valve (Figure 1). The anomalous vessel followed the expected course of the left coronary system along the left atrioventricular groove. The left main coronary artery measured 3.2 mm in diameter, terminating into the left anterior descending and circumflex arteries. The right coronary artery was markedly dilated, measuring 6.8 mm at its mid-segment, with prominent tortuosity and extensive collateral vessels communicating with the distal left coronary system, consistent with the adult or collateral-dependent subtype of ALCAPA [5]. No significant atherosclerotic stenosis was identified in either coronary system. The aorta and remaining thoracic vasculature were unremarkable [6]. However, individualized treatment decisions must carefully weigh operative risks against anticipated benefits, particularly in elderly patients with adequate collateralization who demonstrate stable symptomatology [7]. Recent reports of conservatively managed elderly ALCAPA patients have demonstrated acceptable mid-term outcomes with diligent surveillance. This case highlights several clinically significant aspects. First, ALCAPA survival into the seventh decade of life is exceedingly rare, with fewer than 20 cases reported in patients over 65 years of age in the contemporary literature. Second, this case demonstrates the diagnostic utility of CCTA with advanced post-processing techniques in establishing the diagnosis non-invasively. CPR provides precise anatomic detail regarding vessel origin, course, and collateral status, while cVRT offers photorealistic three-dimensional visualization that enhances spatial understanding of complex anomalous anatomy and facilitates effective communication among clinicians, surgeons, and patients [8]. Third, this case illustrates that conservative management with pharmacologic therapy and close surveillance may represent a reasonable strategy in carefully selected elderly patients with adequate collateral circulation, emphasizing the importance of individualized therapeutic decision-making based on comprehensive multimodality assessment [9].

## Data Availability

No new data were created or analyzed in this study.

## Abbreviations

The following abbreviations are used in this manuscript:

ALCAPA	Anomalous left coronary artery from the pulmonary artery
AO	Aorta
CCTA	Coronary computed tomography angiography
CPR	Curved planar reformation
cVRT	Cinematic volume rendering technique
LAD	Left anterior descending
LCA	Left coronary artery
LCX	Left circumflex
MPA	Main pulmonary artery
RCA	Right coronary artery

## References

[1] Blickenstaff E.A., Smith S.D., Cetta F., Connolly H.M., Majdalany D.S. (2023). Anomalous Left Coronary Artery from the Pulmonary Artery: How to Diagnose and Treat. J. Pers. Med..

[2] Talkhatova S., Aripov M., Mussayev A., Alimbayev S., Otarbayev Y., Pya Y. (2023). ALCAPA in adult asymptomatic patient: A case report. Int. J. Surg. Case Rep..

[3] Suchodolski A., Królikowska M., Kowal A., Głowacki J., Szulik M. (2026). Anomalous left coronary artery from the pulmonary artery in adults: A systematic review of clinical presentation, diagnosis, and outcomes. Int. J. Cardiovasc. Imaging.

[4] de Stefano D., Bitonti M.T., Vertulli D., Zobel B.B. (2024). A case of “late adult type” of ALCAPA syndrome in a 76-year-old woman. J. Cardiol. Cases.

[5] Ojha V., Pandey N.N., Kumar S., Ramakrishnan S., Jagia P. (2021). Anomalous origin of left main coronary artery from pulmonary artery: Patient characteristics and imaging associations on multidetector computed tomography angiography. J. Card. Surg..

[6] Liu Q., Jiang J. (2023). Cinematic rendering of the coronary-pulmonary arterial fistula. Radiol. Case Rep..

[7] Wang J., Hu P., Qiu C.T., Ma X.-J., Xie J. (2025). Comparative Analysis of Coronary Artery Anomalies Originating From the Pulmonary Artery: Clinical Presentation, Imaging Findings, and Surgical Outcomes in Diverse Age Groups. Heart Lung Circ..

[8] Jöbstl A., Wirth L., Feuchtner G.M., Mair J., Widmann G. (2025). The patient’s decision dilemma after screening coronary computed tomography angiography-adult-type ALCAPA with a multimodality imaging approach: A case report. Eur. Heart J. Case Rep..

[9] Duan A., Huang Z., Zhao Z., Luo Q., Liu Z. (2025). Pulmonary hypertension associated with anomalous left coronary artery originating from the pulmonary artery. ESC Heart Fail..

